# Evolutionary Strategies for Body Size

**DOI:** 10.3389/fendo.2020.00107

**Published:** 2020-03-10

**Authors:** Michael A. Little

**Affiliations:** Department of Anthropology, Binghamton University, State University of New York, Binghamton, NY, United States

**Keywords:** human body size, height, human evolution, growth and development, heredity, life history

## Abstract

Humans show marked variation in body size around the world, both within and among populations. At present, the tallest people in the world are from the Netherlands and the Balkan countries, while the shortest populations are central African Pygmies. There are genetic, genetic plasticity, developmental, and environmental bases for size variation in *Homo sapiens* from the recent past and the present. Early populations of *Homo* species also have shown considerable size variation. Populations from the present and the past are also marked by sexual dimorphism, which, itself, shows group variation. There is abundant evidence for the effects of limited food and disease on human growth and resultant adult body size. This environmental influence has been reflected in “secular trends” (over a span of years) in growth and adult size from socioeconomic prosperity or poverty (availability of resources). Selective and evolutionary advantages of small or large body size also have been documented. Heritability for human height is relatively great with current genome-wide association studies (GWAS) identifying hundreds of genes leading to causes of growth and adult size variation. There are also endocrinological pathways limiting growth. An example is the reduced tissue sensitivity to human growth hormone (HGH) and insulin-like growth factor (IGF-1) in Philippine and African hunter-gatherer populations. In several short-statured hunter-gatherer populations (Asian, African, and South American), it has been hypothesized that short life expectancy has selected for early maturity and truncated growth to enhance fertility. Some island populations of humans and other mammals are thought to have been selected for small size because of limited resources, especially protein. The high-protein content of milk as a staple food may contribute to tall stature in East African pastoral peoples. These and other evolutionary questions linked to life history, male competition, reproduction, and mobility are explored in this paper.

Human adult body size shows remarkable variation. In evolutionary and biogeographical contexts, this almost certainly results from the widespread distribution of human populations across the globe, their exposure to varying environments, evolutionary forces, and from complex forms of cultural behavior. In addition to non-cultural environmental selection, human culture diversity can lead to limited gene flow and population isolation, genetic drift, variable selection, and other factors that contribute to human biological diversity and complexity. Hence, human adaptation to the environment entails both cultural and other environmental selective forces that have contributed not only to population variation in body size and morphology, but to all of the uniquely-human attributes that we see in our present species. In addition to the fundamental evolutionary forces of mutation, genetic drift, gene flow, and selection leading to genetic variation, there are the factors of developmental adaptation and genetic plasticity that constitute the total adaptive “package.” In body size and morphology, the adult human is thus an adaptive summation of developmental steps toward maturity—each of the steps in response to the developmental environments acting via the genome through time. Evolutionary strategies involving section may then act through growth and development or be targeted toward maturity and adulthood with important survival and reproductive outcomes.

## Growth to Adulthood

**Figure 1:**
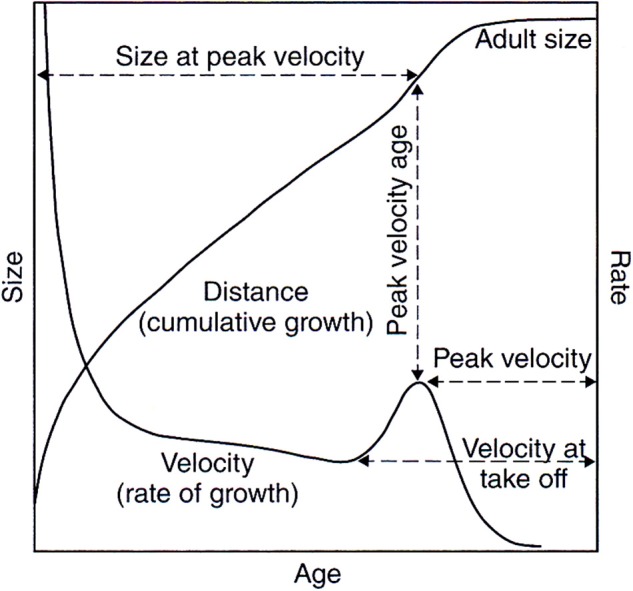
Distance and velocity growth curves that show the generalized human pattern of growth from birth to maturity. Girls are slightly smaller than boys at all ages until adolescence that is 2 years earlier than boys. Girls' peak velocity is less than boys and they reach maturity about 2 years earlier than boys.

Humans are born after a 38-week gestation in what is known as an *altricial* state; that is, the newborn is helpless and totally dependent on the mother for nutrition, warmth, and protection (1, 2). This state persists through infancy and early childhood, during which time motor and perceptual skills, feeding, and cognition continue to develop. Infant growth in size, which is very rapid during the first year, begins to slow down, while brain growth only slows by ages 7 or 8 years, at which time a mature brain weight (but not brain function) is achieved (3). Some degree of independence is reached by 7 or 8 years during this childhood period of steady, but relatively slow, growth in height. Puberty and adolescence are marked by a rapid growth spurt and the beginning of sexual maturation. In girls from developed societies, adolescence begins, on average, at age 10 years and in boys, at age 12 years. Sexual and size maturation are achieved by 16 years in girls and 18 years in boys (see Figure 1). In this generalized figure, size or distance is the accumulation of growth rates (velocity) at different ages; or as Franz Boas (4) first described them, different “tempos of growth.” Also, differences in rates of growth of specific structures (e.g., brain, lower limbs) will produce allometric changes in proportions and size at different ages. Therefore, any hereditary or environmental influence on growth rate at a given age, particularly infancy or adolescence when linear growth rates are high, can enhance or retard adult size. There is considerable variation in these ages of growth and maturation in populations throughout the world, and a number of steps in this trajectory where growth can be enhanced or retarded. Adult body size results from the cumulative growth at each of the stages (fetal, infancy, childhood, adolescence). Recent research has suggested that the prolonged growth to human maturity is a trade-off between the energy needs to fuel somatic (body) growth vs. the energy needs of the large growing brain. Kuzawa et al. (5) showed that peak human brain glucose demands occur during childhood (ages 4–10 years) when somatic growth is slow and prolonged; hence, allowing greater amounts of energy to be devoted to the rapid growth rate of the brain.

Enhanced fetal growth is limited by the size of the maternal pelvic birth canal, but large women who have large pelves are capable of delivering larger (>3,500 g) full-term fetuses. Hence, selection for human birth size is linked to maternal pelvic size and body size, a form of co-evolution that occurred as a part of human fetal evolution. There is, of course, a co-dependency between the mother and fetus during gestation that is based on both the maternal and fetal genomes and the maternal intrauterine environment. Smaller than average mothers tend to deliver the fetus at a slightly earlier gestational age and have smaller infants (6) and there is a clear relationship between birth weight and length and adult weight and height (7–9). Birth order also has an influence on height of children, where later parity children are successively shorter than early parity children (10). Meredith (11) compiled birth weights for different populations around the world. The average range of variation was between 2.4 kg in the New Guinea Lumi (an average below the “low birth weight” cutoff of 2.5 kg) and 3.6 kg in United States Native Americans. In addition to suggested ethnic differences, there were a host of likely socioeconomic, environmental, and health influences.

Both genetic and environmental variations play a role in structuring growth during childhood and adolescence, and in complex ways. For example, during adolescence, the onset, duration, intensity, and completion of the adolescent growth spurt (marked by velocity changes) can affect adult height. Late maturers begin adolescent growth at a slightly taller height than early maturers, which partly contributes to the greater height of adult males over adult females. Other factors contributing to height differences between the sexes are the slightly greater size of boys than girls at birth that persists throughout infancy and childhood, and the substantially greater amplitude in boys of the adolescent growth spurt when compared with girls (12). Despite these relationships, the correlation between early and late maturation and adult size is weak and it is possible for both early and late maturers to reach the same stature (13).

At all prenatal and postnatal ages, the primary drivers and regulators of growth are nutrient and energy intake and hormones (14–17). Variations in the output of human growth hormone (HG), insulin-like growth factors (IGF-I, IGF-II), insulin-like growth factor binding proteins (IGFBP), and other hormones, and the metabolic pathways leading to tissue receptivity, contribute to human growth within the parameters of the fundamental human growth curve (18). Evolutionary changes in the genetic control of these growth regulatory pathways can produce changes in the duration or velocity of growth at any age and can produce allometric changes and changes in overall body size. An excellent review of these regulatory hormonal pathways and their influence on human size during growth and development and through evolutionary time is found in (19).

Environmental influences on growth to adulthood can produce a range in variation in stature within a given population, but any single population does not display the full range in variation expressed in human populations throughout the world. Hence, in addition to environmental influences on stature, genetic, and epigenetic factors are shown when population averages are compared. These population differences will be discussed below.

## Human Population Variations in Body Size

### Our Hominin Ancestors

Within a given taxonomic category (such as *Hominini*, the taxonomic Tribe or evolutionary clade that includes all members of the genera *Australopithecus* and *Homo*), there are often larger and smaller body-sized representatives of species and of populations within species. In an evolutionary context, there are both selective advantages to being small and selective advantages to being large (20). Large body size tends to be associated with higher fecundity, reproductive success in male vs. male competition, greater protection against predators, and ability to combat cold climates. These advantages are countered by selection against large body size from a number of pressures. For example, viability costs are great when the time to sexual maturation increases (greater cumulative mortality) and are less with rapid growth and shorter maturation time. Also, larger bodies increase metabolic needs and intensification of food acquisition (with associated risks) and are linked to size-selective hunger and starvation. Finally, heat stress in hot climates or with physical activity is intensified with larger bodies and there may be reduced agility or increased detectablity (20, 21). In these cases, selection operates through differential survival or mortality and differential fecundity/fertility and reproduction, and a probable equilibrium in size is achieved according to any given set of ecological conditions under which a species or population is living. These principles have been identified and tested for invertebrate and vertebrate species, but apply to human species, as well (22, 23).

Ruff (24) identified several patterns of body size and shape variation in our Pliocene and Pleistocene hominin ancestors. In the transition from *the* Australopithecines to early *Homo* (*Homo habilis*/*rudolfensis*) about 2 to 2 1/2 million years ago, and the later species, *H. erectus*/*ergaster*, after 2 million years ago, there were significant increases in body size, brain size, and allometric form of the limbs. Australopithecine species body mass ranged from about 35 kg to 50+ kg, with the more robust species at the higher range. Early *Homo* body mass ranged from about 35 to 70 kg and was highly variable in stature (between 145 and 184 cm; similar to a modern population range). Shifts in locomotion, foraging, and diet were suggested as some of the selective pressures leading to these changes.

The second transition was around 500,000 years ago with a further increase in body size (mass) that Ruff (24) attributed to hominin populations moving to higher latitudes and colder climates. The estimated body mass of these mid-Pleistocene specimens ranged broadly from about 50 to 90 kg. An unusual exception to this is the diminutive *Homo floresiensis*, who lived on the tropical Flores Island (Indonesia) about 80,000 years ago (Late Pleistocene), and who had a stature of about 106 cm and a body weight of about 30 kg. Also, a more recent Late Pleistocene discovery from the Philippines (Callao Cave, Luzon Island) was identified as a new species, *Homo luzonensis*, and thought to be as diminutive as the Flores Island population (25). The third transition occurred about 50,000 years ago in the late Pleistocene, in which there was a decline in body size (mass), particularly among higher-latitude (northern) populations, perhaps associated with increased technology (tool production) and cultural factors that led to less male-male competition.

A fourth transition (Neolithic Revolution) about 10,000 years ago led to a further reduction in body size with the shift from a hunting-gathering (foraging) subsistence pattern to a food production pattern. In addition to dietary changes, technological advancements may have contributed to this latter transition. It should be noted, however, that with the Neolithic and later dominance of cultivation and animal domestication, many populations continued foraging subsistence patterns up to the present.

A more recent interpretation of body size variation in our early ancestors arose from a Wenner-Gren Foundation symposium in 2011 (26). They noted that smaller-bodied versions of *Homo erectus* have been found in Kenya and the Republic of Georgia, and the diversity in other populations of *Homo* has been less appreciated, perhaps, in an emphasis to characterize *stages* of evolution. This newer approach to understanding human evolution has centered on the variation in forms of this genus in response to “shifting environments” and how early *Homo* can be better understood in the context of “how extant humans, non-human primates, and social carnivores respond energetically, physiologically, and socially to changes in resource availability and to stress from climatic, environmental, and other factors” [(26), p. S270]. In addition, Kuzawa and Bragg (27) concluded that developmental plasticity contributes to considerable human variation in contemporary populations, and it is assumed, then, that corresponding plasticity has contributed to variability in past populations, as well. Also, since selection operates on the phenotypic expression of developmental plasticity, then environmental modification of human biology and behavior is a major evolutionary force.

### Living and Recent Populations

We know a great deal about body size and morphology of both adult and young members of contemporary human populations. The compilations of anthropometric measurements taken around the world during the late 19th and 20th centuries, and particularly those taken during the International Biological Programme (IBP) surveys from the Human Adaptability research (28), have been summarized by Eveleth and Tanner (29, 30) according to ethnicity, geographic location, and migration patterns. These measures of height, weight and different body proportions show marked variation, both within and among populations. The studies compiled by Eveleth and Tanner (29, 30) tend to describe numerically small populations, worldwide, with indications of variation listed in appended tables.

More recent worldwide compilations of body size in stature are found in Bentham et al. (31) and Roser et al. (32). These two global works are extensive, but the data are limited because reporting is done according to larger population units (nations and regions), and much of the population-specific human variation is lost. For example, Roser et al. (32) list the shortest men on record at 160 cm in stature from Timor in island Southeast Asia, whereas Cavalli-Sforza (33) and Dietz et al. (34) identify Congo men with the pygmy phenotype (short stature; usually <155 cm in men and 150 cm in women) from the Ituri Forest as averaging slightly <145 cm tall, a difference of 15 cm. However, despite the large population units surveyed, these two latter global reports are quite valuable because of the long-term data collected on stature changes according to birth cohorts. The Bentham et al. (31) compilation (NCD-RisC) provided data on mean adult heights of birth cohorts from 1896 to 1996. These data then allow global analyses of what is known as “secular changes” in growth and maturation; that is, those increases or decreases in body size over the short-term that are largely a function of environmental changes in health and nutrition affecting growth to adulthood [(35, 36), p. 116].

If we consider African men with the pygmy phenotype at an average stature of 145 cm and pygmy woman at a stature of 136 cm as the shortest contemporary populations on record (33), and the tallest as the average Netherlands' man at 184 cm and the average Netherlands' woman at 171 cm (37), then the mean difference in the population average range for our species is a remarkable 39 cm for men and 35 cm for women! Other European nations with very tall populations include Bosnia, Herzegovina, and Croatia, where average young men from a widespread survey of these three Balkan countries ranged in height from 180 to more than 184 cm (38, 39). In addition to the African pygmy phenotype, other short-statured populations, largely foraging or horticulturalist peoples, can be found in enclaves in South America, Southeast Asia, Papua-New Guinea, and equatorial Africa. Table 1 lists several of these populations with recorded heights and weights. Many of the shortest-statured populations are from either tropical forest environments or tropical islands. Exceptions are the Ju'/hoansi San and !Kung San populations (formerly Bushmen) from semi-arid lands in southern Africa, but these populations are at the higher range of short-statured peoples. Another population at the higher range of heights is the Twa, from the Lake Tumba region of the Democratic Republic of the Congo, who are highly admixed with neighboring Bantu farmers (50).

**Table 1:** Heights and weights of several short-statured foraging and horticultural populations.

	**Males**		**Females**		
**Population**	**Ht (cm)**	**Wt (kg)**	**Ht (cm)**	**Wt (kg)**	**References**
Aché (Paraguay)	158	60	149	54	(40)
Aeta (Philippines)	150	40	140	38	(41)
Agta (Philippines)	153	45	144	38	(42)
Batak (Sumatra)	153	47	143	41	(43)
Bundi (Papua New Guinea)	150	52	146	–	(44)
Hiwi (Venezuela)	154	56	145	48	(45)
Ju'/hoansi (Botswana, Namibia)	159	55	149	50	(46)
!Kung (Botswana, Namibia)	160	49	150	41	(47)
Onge (Andaman Islands)	148	–	138	–	(48)
Pygmy (Efe, Ituri Forest, Congo)	143	43	136	38	(49)
Pygmy (Mbuti, CentralAfrican Republic)	144	42	136	37	(33)
Pygmy (Twa, Congo)	160	46	150	42	(50, 51)
Yanomamo (Venezuela, Brazil)	152	52	142	45	(52)

Within these extremes of height from the shortest to the tallest populations, there is considerable variation by geography, ethnicity, nationality, and socioeconomic levels. Roser et al. (32) listed global averages of the height of men born in 1996 as 171 cm and women from the same cohort as 159 cm. The average sexual dimorphism in height is thus about 12 cm, although sexual dimorphism varies substantially for populations around the world. These authors identify the shortest men and women from South Asia and the tallest from Europe and Central Asia, although there are exceptions to these generalizations in each of these large geographic areas. In sub-Saharan Africa, for example, there are many short-statured populations (e.g., Pygmies and San), yet some of the East African populations are quite tall (e.g., Maasai and Turkana) (53, 54). NHANES data from 2007–2010 in the United States provides some reference statistics to demonstrate ethnic (and probably socioeconomic) variation in height for a large national population (55). Young men and women aged 20–39 years were listed as Non-Hispanic White, Non-Hispanic Black, and Hispanic. The White American group's heights for men and women were 178 and 165 cm, respectively; the Black American group's heights were 176 and 164 cm; and the Hispanic group's heights were 171 and 158 cm. These ethnic differences reflect just some of the variability found in any national or large-scale population.

### Environmental Factors in Body Size Variation

The variation displayed in this single variable, height, in contemporary populations is based on numerous influences from the environment, genetics (the genome and gene pool), and the genetic plasticity tied to the interaction of genes and environment. In many cases, it is easier to demonstrate environmental than genetic causality. Major environmental factors that can lead to developmental variations in body size are diet and nutrition, disease history, general sanitation and clean water availability, infant and child care, and cultural practices that can influence all of these other variables. Each of these environmental factors will contribute to human health, where optimizing health throughout the period of growth and development to maturity will lead to an optimal body size according to individual genome constitutions.

### Diet, Nutrition, and Disease

**Figure 2:**
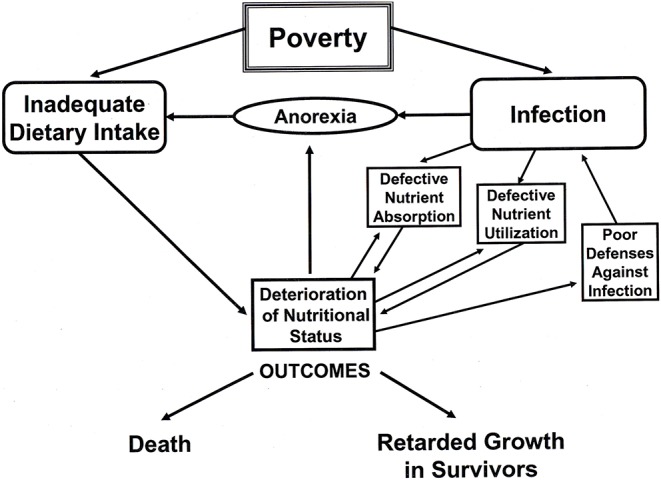
A diagram illustrating the cascade of effects of poverty, such as limited food resources, infectious diseases, and poor sanitation. Redrawn from a diagram by Martorell (61).

Human societies/populations have highly varied diets and cuisines, and it is a characteristic of humans that they have a broad adaptive capacity for dietary diversity. However, not all diets are optimal for human growth, and food intake variations can have a profound influence on general health and body size at all ages. We know, also, that human diets are not always stable and there are periods of hunger and dietary deficiencies linked to climatic, political, economic, and other factors. For example, at an early stage of development, maternal malnutrition will negatively impact fetal growth (intrauterine growth restriction, IUGR) and may have long-term impacts leading to metabolic health disorders in middle age (56, 57). Acute respiratory and gastrointestinal disorders in children can also have profound negative effects on growth, particularly in infancy and early childhood (58–60). Poverty or limited access to resources can lead to inadequate dietary intake, disease, and anorexia, all of which will result in stunted growth and small body size in adults (61, 62). A cascade of effects of poverty leading to stunted growth (>2.0 SD below the mean of WHO standards) is diagrammatically and dramatically illustrated in Figure 2. Another indirect cause of disease in the lower income countries is poor sanitation in densely-populated areas, also linked to poverty. For example, Spears (63) demonstrated a strong correlation in India between open defecation (with a lack of toilets or latrines and presumed spread of fecal pathogens) and stunting in children. The average child fell 2.0 SD below the mean for child height when only 20 percent of the population had access to toilets or latrines.

Quantitative and qualitative protein intakes are important in contributing to stature variation in humans (64). A qualitatively important protein source is cow's milk, a staple food in many Western societies. Although milk is an excellent source of protein and calcium, it also contains lactose, a milk sugar that cannot be digested by many adult peoples around the world. Most native peoples from Asia, West Africa, the Pacific Islands, Australia, and the New World are unable to produce the intestinal enzyme, lactase, to break down the milk sugar, lactose, into its constituent (and digestible) monosaccharide sugars, galactose and glucose. In many Europeans, populations from the Near East, and East African herding peoples, the ability to digest lactose as adults is under genetic control; hence, selection has operated in favor of “lactase persistence” in these milk-producing populations. Tishkoff et al. (65) found that independent selection for different genetic systems produced convergent evolution for African pastoral and European people's genetic systems. It is also the case that, in addition to the value of milk as a dietary item, milk intake beyond infancy and weaning, may contribute to increased height in some populations. Cow's milk has as much as three times the protein content and four times the calcium content of human breast milk (although with some variation), and there is evidence that cow's milk consumption contributes to increased height if consumed after weaning and during late childhood and adolescence (66, 67).

A case in point is East African pastoralists, who are tall and lean in physique, and who consume significant amounts of milk in their diets as a staple food (68). Using the Turkana nomads of northwest Kenya as an example, cow's, goat's, camel's, and sheep's milk constitute as much as 80 percent of the diet during the wet seasons when forage is abundant and livestock milk production is high (69). Because of the availability of animal milk, Turkana women, who breastfeed their infants for an average of 20 months, begin to supplement infants' diets with animal milk at 5–6 months of age (70). This is consistent with the pattern described by Wiley (67) that contributes to accelerated height in other populations in later years. At 6 months of age, Turkana infants begin faltering in weight (behind Western norms), but length is equivalent to U.S. standards. Although adolescent growth is not extraordinary, growth in stature is attenuated and continues into the early 20s, such that adult height by age 23 or 24 years in both men (174 cm) and women (161 cm) is close to U.S. African-American standards (54). Hence, despite the Turkana experiencing seasonal and other short-term bouts of hunger, and with average daily energy intakes well below international standards, but protein intakes at 3- or 4-times minimum daily requirements, they are able to achieve tall statures that are substantially greater than non-pastoral native populations in Africa and other parts of the lower income countries. They are, however, extremely lean in physique, with young adult body mass indices (BMI) between 17.0 and 18.5 kg/M^2^ (71).

### Socioeconomics and Culture

Socioeconomic status can be defined by a complex variety of factors including family history, social position, role definition, ascribed and achieved status, occupation, material wealth, and other variables. Different levels of socioeconomic status carry with them a variety of health advantages and disadvantages that are a part of the life experience as noted above. Social mobility can influence some of the factors (72) related to health status, but within-generation mobility cannot transform life history.

Influences of socioeconomic class on body size were demonstrated in a study of Scottish children aged 11 years in the early 1950s [Tanner (73), p. 138]. A gradient existed where children of professional class fathers were about 3 cm taller and 1–2 kg heavier than children of manual workers. Lasker and Mascie-Taylor (74) based their study on data drawn from the National Child Development Study of all children in England, Scotland and Wales during the 3rd−9th of March 1958. Longitudinal follow-up studies were conducted of children at 7, 11, and 16 years of age on a sample size of ~16,000. Social class designations were based on the occupation of the male head of household (highest occupational class = professionals, lowest occupational class = unskilled laborers). Differences in height between children from difference occupational classes were achieved by 7 years of age and very little height differential was acquired after that age. Mascie-Taylor (72) showed that upwardly mobile children were smaller than their next higher class but larger than the previous socioeconomic class. Social mobility, like geographic mobility or migration (75), can contribute to body size differences both within and accross generations (76).

### Secular Trends in Growth and Maturation

*Secular trends* in human growth are short-term trends (years, decades, and transgenerational periods) in growth patterns, proportions, and maturation. The assumption is that a secular trend, even over 100 years or more, is reversible, and hence, is largely a function of environmental, sociocultural, and general health conditions. In an early study of age cohorts of children from the Horace Mann School of Columbia University, Franz Boas (35, 77) found that children had become taller between 1909 and 1935. He attributed these changes to improvements in socioeconomic conditions leading to a modification of the “tempo of development,” that is, the rate of growth of the children. This phenomenon has been documented repeatedly since that time and has been referred to as “secular trends in growth” [(73), p. 143–155, (36), p. 116, (78)]. Secular trends can be either positive (e.g., increases in size, earlier maturation) or negative (e.g., decreases in size, later maturation). In the former case, improved nutrition and health are the proximate causes, while in the latter case, a downturn in or worsening of health and nutrition are the principal causes. Because the time period is relatively short, it is unlikely that these changes in growth, maturation, and adult sizes are evolutionary changes. Economic upswings and downswings are reflected in corresponding conditions of relative health and welfare; for example, as existed before, during, and after the great depression in the early half of the 20th century.

A marked secular trend in Western nations began in the mid-18th century with the Industrial Revolution and economic improvements for workers and their families. In addition to increases in average heights of children at all ages during the 19th century, there were dramatic declines in age of menarche from 17 years of age in 1800 to 13 years of age in 1960 (earlier reproductive maturation) [(73), p. 153]. This trend has continued in contemporary populations undergoing economic expansion and experiencing economic prosperity (79). For boys, Daw (80) demonstrated a secular trend in voice change associated with puberty in J.S. Bach's early- to mid-18th century Leipzig choristers, where choir boys experienced voice changes around 17 or 18 years of age in contrast to modern choir boys who show voice changes at about 13 or 14 years of age. Although there are no records of height for Bach's choristers, late maturation was likely to be linked to short stature. Another good example is the record of heights of boys who were recruits at the Royal Military Academy at Sandhurst and the Marine Society of London between 1750 and 1950 (most were poor boys aged 13–18 years) (81). There were marked differences by social class and height changes through time that reflected secular trends in economic prosperity and life styles. For example, 15-year-olds who were 140 cm tall in 1750, were nearly 30 cm taller at the same age in 1950. This displayed a generally positive secular trend in height over that 200-year period (but with several short negative trends due to economic downturns in the late 1700s and mid-1800s).

Another major episode of secular changes in growth and adult size occurred after World War II in Europe (31, 82). The result of this post-WW II secular trend in height was the genesis of some of the tallest people in the world from the Netherlands and the Balkan States (as noted above). This trend appears to have slowed or stopped at present, perhaps reaching, what Cameron (78) has called, a “genetic ceiling.”

## Genetic and Evolutionary Factors in Body Size Variation

There are abundant examples of environmental, socioeconomic, cultural, and health influences on body size and stature/height. But what evidence do we have for hereditary and possible evolutionary, selective, and adaptive factors associated with body size?

Human height is a complex, quantitative variable that is based on cumulative increments of growth over a prenatal and postnatal maturational period of up to 20 or more years. It is a useful variable for hereditary analysis because there is a wealth of data that have been collected over the years. The variable is polygenic and involves multiple genetic loci with punctuated genetic expression over this maturational period. Although the heritability of height is great [*h*^2^ = 0.80, (83)], height is also subject to substantial environmental modification and plasticity; hence, identifying the genetics of height has been difficult using traditional methods such as family and twin analysis.

### Genome-Wide Association Studies (GWAS)

More recent work has included genome-wide association studies (GWAS) and meta-analyses combining data from a number of independent studies to provide more detailed data on height-associated genetic loci and single nucleotide polymorphisms (SNPs), some of which may be quite rare. The earliest GWAS studies discovered 47 loci with SNPs associated with height (83). Most recent meta-analyses have found hundreds of loci with several thousand SNPs that are connected to biological pathways, in turn, associated with adult height and growth factors (84, 85). The SNPs associated with human growth are linked to chondrocyte proliferation and differentiation, growth plate formation, and bone development (83, 86), and later growth spurt in males and later puberty in females (87).

While much of the research has been conducted on living European populations (88, 89), some research has been done with ancient DNA derived from skeletal remains (90). Cox et al. (90) observed a decrease in stature between Early Upper Paleolithic and Mesolithic and Neolithic peoples (transition from hunting and gathering subsistence to early farming and herding), but an increase in height between the Neolithic and the Bronze Age (about 5,000 years ago). They also found that higher geographic latitude peoples were taller than lower latitude peoples. Finally they stated that with all height-associated genetic variants combined, it is now possible to predict about 30 percent of the phenotypic variance in the height variable.

### Evolutionary Processes and Strategies

A productive way to study evolutionary strategies of human body size variation is through life history theory (27, 91, 92). Life history studies attempt to answer questions about evolutionary processes leading to the origins of and variations in human attributes, such as: body size; timing and events of growth to maturity; fertility and reproduction; mortality; energy allocation to growth, maintenance, reproduction, and longevity; and all in the context of the human life cycle. Energy allocations to (1) growth, (2) maintenance, (3) reproduction, and (4) longevity (= reduced mortality), require evolutionary trade-offs or compromises through natural selection for favorable outcomes. The question to be explored for human body size is: What is a favorable body size (or body sizes) to optimize these four main variables considering evolutionary trade-offs?

**Figure 3:**
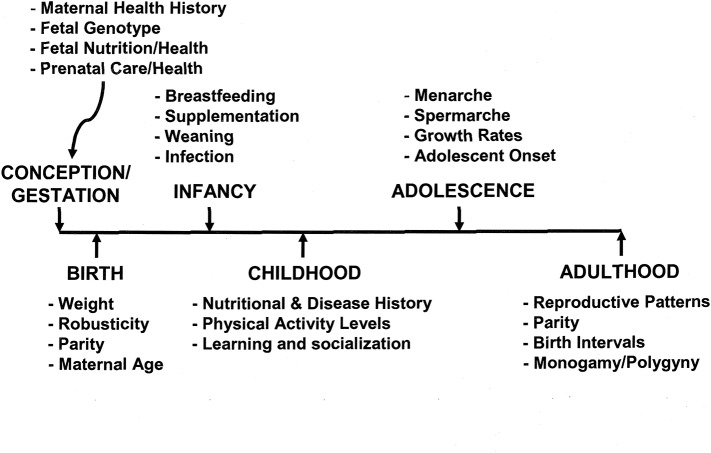
A time line from conception to adulthood of life history, illustrating some of the crucial variables that influence adult size and, potentially, reproduction.

Figure 3 illustrates some of the variables to be considered in analyzing the human life cycle and individual life histories. Selection operates on body size from conception to maturity, where adequate nutrition and relative absence of infection will allow for optimal growth. However, optimal growth depends on the mix of food resources, exposure to disease, and a host of other variables—environmental, human biological, and cultural. For example, Blanckenhorn [(20), p. 385–6] noted that “It is widely agreed that fecundity selection in females [for optimal fertility] and sexual selection in males [for mate competition] are the major evolutionary forces that select for larger body size in many organisms.” For humans, larger women have larger newborn infants and larger infants have a higher survival rate than smaller infants. However, the energetic (or caloric) costs of maintaining a larger female, gestating a larger infant, and breastfeeding this infant for a year or more are substantial and may not be possible in a subsistence society. A case in point is the short-statured KhoeSan peoples of southern Africa. Based on skeletal remains from the Later Stone Age and contemporary body size values, Pfeiffer et al. (93) speculated on selective pressures that acted on these populations from the Later Stone Age up to historic times when Bantu and European intrusions probably modified the ecological and cultural conditions of the KhoeSan peoples. There are advantages to a shortened growth period and smaller size in organisms because the reduced time to sexual maturity allows less time for pre-adult mortality (viability selection) (20). However, there is no evidence for reduced maturation time in KhoeSan populations. Sociocultural factors, including reduced male to male competition, mobility needs, and a dispersed food supply were suggested as selective pressures leading to small body size. Another example of sexual selection playing no role in a short-statured population is for a central African pygmy population (94). Baka pygmy couples were compared with Nzimé Bantu couples, where they were tested on preference for short or tall partners. The authors found that there was positive assortative mating for stature in both groups of couples, but for the Pygmies, the assortative mating actually led to a slight preference for tallness.

An example of food energy availability and births can be given for Turkana pastoralist women, who are lean in physique, but have a moderate-to-high completed fertility (CF = 7 live births). Nevertheless, because of limited food resources unique to their cultural subsistence practices, the women lose body energy stores with subsequent pregnancies placing late parity infants at higher risk than early parity infants (95). Also, breast feeding patterns are linked to maternal size, where larger (taller and more robust) mothers had a greater frequency of breast feeding bouts than thinner mothers (96). The trade-offs occur in women's body size and the ultimate fertility outcome along with infant and child mortality rates. On the other hand, selection for smaller body size in mothers is energetically less costly, but produces smaller newborn infants who are at higher risk of morbidity or mortality due to poorer temperature regulation, less effective protection against dehydration, and greater fragility (97, 98). Yet in this case, selection for smaller female body size may allow for a higher overall fertility and justify the outcome via higher fitness.

### Short-Statured, Pygmy, and Pygmoid Populations

Living populations with individuals of very small body size are rare, and are of particular interest within the framework of life history and evolution. These populations (see Table 1) are characterized generally as remnants of hunter-gatherer or foraging populations who are either tropical-island or tropical-forest residents. They are somewhat isolated and often inhabit ecosystems with limited food resources. For Africans with the pygmy phenotype and other “pygmoid” populations, there are several selective pressures that have been hypothesized to explain the short stature of these peoples, that is, relative to the taller stature of a majority of living human populations (99–101).

One hypothesis bears on temperature regulation, in that short stature tends to maximize surface area per body weight relationships and should give pygmies an advantage in hot-humid tropical forests. Pygmy short stature is consistent with Bergmann's rule that a small body with high surface area to volume ratio is effective at dissipating body heat in the tropics, particularly the humid tropics. With elevated environmental humidity, evaporative cooling (sweating) is a less effective way to dissipate body heat, but a higher surface area should provide a slight advantage through passive convective heat loss. Austin and Ghesquiere (102) and Austin and Lansing (103) tested Bergmann's rule with temperature stress tests and modeling on Twa pygmies in the Lake Tumba region of the Democratic Republic of the Congo but with limited results; however, on theoretical grounds a greater surface area to weight ratio should provide a slight selective advantage under hot-wet conditions (see *Biogeographic Rules* below). A second hypothesis suggests that small body size is advantageous in reducing individual and population food energy requirements. Small bodies are metabolically efficient and a response to limited food and protein resources is often an evolutionary reduction in body size. This can be the case with pygmy mammalian species found on island or population enclaves – sometimes referred to as “insular dwarfism” (104). Bailey et al. (105) argued that pygmies have traditionally participated in reciprocal exchanges of food (“symbiotic” relationships) with their food-cultivating neighbors because tropical rainforests have limited energy-rich food resources. However, Bahuchet et al. (106) have largely dispelled this myth by demonstrating that the presence of wild yams and other food plants in tropical forests would have allowed early rainforests to have been exploited by foragers before the onset of food cultivation. A third hypothesis has been suggested based on human mobility and agility, such that dense jungle can be more easily navigated by small-sized individuals (101, 107). Navigating dense undergrowth and accessing resources in which tree climbing is a favored skill might have selected for small body size. These are difficult hypotheses to test and the real answer may be in some combination of or all of the causes.

Studies of pygmy growth and development have uncovered endocrine and genetic causes for their phenotypically short stature. Merimee and Remoin (108) gathered data on insulin-induced human growth hormone (HGH) and arginine-induced human growth hormone and associated hormones from 22 pygmies in the Central African Republic. For both tests they found no differences in HGH levels between the pygmies and a European control group; however, the pygmies' plasma glucose responses to insulin and their plasma insulin responses to arginine were similar to HGH-deficient dwarfs. Further studies by Merimee and Remoin (108) demonstrated that pygmies had peripheral tissue insensitivity to HGH due to a deficiency of somatomedin or insulin-like growth factor I (IGF_I_), a hormone which affects skeletal growth. Recent work by Bozzola et al. (109) has supported these early studies where they found a marked reduction in HGH receptor gene expression and that pygmies lack both an adolescent growth spurt and an associated pubescent serum IGF_I_ surge. Also, Becker et al. (110) found distinct differences in Cameroon Baka pygmies and their neighboring non-pygmy Nzimé populations in growth hormone receptor (GHR) and insulin-like growth factor 1 (IGF1) gene frequencies. Studies of the short-statured Aeta from Luzon in the Philippines were conducted by Bernstein and Dominy (111) to test several bioactive breast milk factors (cytokines) as having potential influence on epigenetic “inflammation memory” (maternal to infant transmission via breast milk) and its possible influence on infant growth. Comparisons with Ilocano Philippine and other populations were inconclusive, but the authors suggested that the Aeta population “…offer promise as a model system for testing epigenetic hypotheses focused on the relationships between adult mortality, age of reproductive maturity, and stature.” [(111), p. 244]. Finally, convincing evidence that short pygmy stature is under genetic control was provided by Becker et al. (100) via admixture studies of more than 1,100 pygmy and Bantu central African adults from Cameroon, the Central African Republic (CAR), and Gabon.

The effects of genetic differences on growth processes appear to differ among pygmy populations, particularly between the Western cluster of populations with the pygmy phenotype (Gabon, Cameroon, and CAR) and the Eastern cluster of the Congo basin (112). In Eastern Congo Efe and Ngayu Pygmies, birth weights (2,600 g) and birth lengths (44 cm) were slightly less than their Bantu neighbors (birth weight = 3,000 g, birth length = 47 cm) (113). Hence, Efe and Nagau pigmy infants began life at smaller sizes than Bantu. In a large longitudinal sample of Western pygmies (Baka) studied over the long term by French CNRS researchers, pygmy birth weights were close to French standards but the pygmies fell behind the French throughout infancy with values reaching −2 standard deviations (−2 Z-scores), and pygmies remained at −2 Z-scores until adulthood (112). Differences have also been found in adolescent growth spurts: some pygmy populations showed suppressed adolescent growth (114) while others displayed normal adolescent growth (112).

Despite these differences in growth patterns in Western and Eastern African pygmy clusters leading to phenotypic short stature, genetic signatures have recently been identified in both African (Ugandan Batwa and Cameroon Baka) and Asian (Andamanese Onge) that are associated with growth factor binding functions and convergent selection in these tropical rainforest hunter gatherers (115, 116). What is quite clear for these and other studies is that pygmy populations have growth patterns that differ from other populations that lead to short stature, and that these growth patterns have a genetic basis that may have involved convergent evolution between African and Asian short-statured populations.

Returning to the selective pressures hypothesized to have acted to produce short stature, a fourth hypothesis, developed more recently than the other three, has centered on life history processes, mortality, and fertility (41, 52, 117, 118). The hypothesis is associated with a life history trade-off between accelerated growth and early sexual maturation as a compensation for high mortality at relatively young and older (adult) ages and a truncated reproductive life span. This was hypothesized by Migliano (41) and Walker et al. (52) and tested by Migliano et al. (117). Both Walker's and Migliano's research employed comparative data from Asian, African, and South American hunter-gatherer (H-G) populations. Walker et al. [(52), p. 295] suggested it is “…selective pressure for accelerated development in the face of higher mortality…” that has led to the short stature of many H-G peoples. Hence, the argument is that short stature is a by-product of this fertility shift, and there is not direct selection for short stature. Within this theoretical framework, Walker et al. (52) explored growth rates in some detail in 22 small-scale, foraging societies in which height and weight velocities could be compared. They found that faster growth (higher velocities) and an earlier puberty were indeed associated with a higher mortality risk and limited food and resources. These conditions of accelerated but shortened growth, then, produced short stature in these populations. An extension of this work comparing small-scale societies (118) incorporated population density and geographic latitude as variables into the mortality and early maturation model producing short stature. Population density was an important variable suggesting enhanced competition for food resources. Correlation analysis demonstrated that increased population density led to decreased probability of survival to age 15 years, and that probability of survival to age 15 years was positively correlated with body size. In other words, high survivorship was related to larger body size and high mortality was related to small body size. Migliano and Guillon (119) compared life expectancy at birth, survivorship to age 15 years, and life expectancy at 15 years with adult height in 89 small-scale human societies. Using linear regression models, they found that adult height variation is strongly predicted by these measures of mortality (and survivorship), such that an accelerated childhood and adolescence will reduce the chances of death before the ages of reproduction. Hence, an earlier reproductive age will potentially increase the reproductive life span and enhance fertility.

There has been criticism of the mortality/fertility/early maturation trade-off hypothesis. Although they encourage research on life history theory and pygmy stature and identify the work by Migliano (41) and Migliano et al. (117) and Walker et al. (52) as innovative, Becker et al. (120) recognized several problems with the trade-off hypothesis. Two criticisms were based on (1) arbitrary height thresholds to distinguish pygmy from non-pygmy populations and (2) mathematical errors in their models. However, the most significant critique centered on pygmy demographic data, where Becker et al. (120) noted that there is insufficient data on age-specific mortality, fertility, and life expectancies for African populations with the pygmy phenotype. This is quite true, but an added problem with the hypothesis is that both fertility and mortality are not constant in most populations. These variables fluctuate with sociocultural, socioeconomic, climatic, resource availability, and a whole host of variables. A case in point is the detailed demographic work done by Leslie et al. (121–123) on East African Turkana pastoralists, where seasonal and annual fluctuations in food resources led to more than 2-fold differences in fertility and marked differences in mortality over the short-term and the long term. More recently and returning to pygmies, Ramirez Rozzi (124) found dramatic declines in Baka fertility that were association with acculturation and the introduction of an alcoholic drink that disrupted family relations in this small-scale society. Another counter-argument to the trade-off hypothesis, then, is that mortality at all ages and age-specific fertility rates are temporally fluctuating variables in all societies, and are unlikely to have remained stationary for sufficient numbers of years for selection to have acted on earlier sexual maturity to compensate for higher early adult mortality.

### Biogeographic Rules

Walker and Hamilton (118) also found a relationship in a large sample of foraging populations to latitude, which is consistent with other literature that demonstrated a gradient from the equator to high latitudes. Shorter individuals tend to be found in populations from lower (equatorial) latitudes (tropical or warm climates) and taller individuals from populations at higher latitudes (temperate or cold climates). This is consistent with Bergmann's (125) biogeographic rule that states that in related species or populations of homeotherms (endothermic vertebrates), those from warmer climates will have smaller bodies than those from colder climates. The rule derives from the relationship between size in linear dimensions (height in humans), surface area, and weight or volume. As the linear dimension increase by one unit, the surface area increases by the square of that unit, and the volume (weight) increases by the cube of that unit. Hence, the important relationship is the surface area per volume or surface area/ weight (SA/wt), where a high SA/wt ratio (short stature) facilitates heat loss from the surface and a low SA/wt ratio tall stature) reduces heat loss from the surface. These relationships are only for passive heat exchange between the body and the environment. There are many physiological mechanisms that control human temperature regulation, but these passive heat exchange relationships have been shown to operate for humans and to influence body size in population around the world (126, 127).

Roberts (126, 128) early studies demonstrated the strong climatic and latitudinal biogeographic relationships with body size for pre-World War II populations. Katzmarzyk and Leonard (127) then verified these relationships for post-World War II populations. However, they found that the correlations were weakened, perhaps with the post-war worldwide spread of Western culture and technology. Ruff (129, 130) extended these comparative studies to prehistoric human populations from skeletal remains. He found that body breadth (from pelvic dimensions) was an equally important variable in regulating SA/wt ratios [a narrower body allowed a higher SA/wt ratio [heat dissipation] and a broad body allowed a lower SA/wt ratio [heat conservation]]. This result demonstrated the corollary of Bergmann's Rule associated with linearity (131), and enabled Ruff (129, 130) to demonstrate the validity of Bergmann's and Allen's rules for the relationships of body size and proportions from skeletal remains for prehistoric populations.

## Discussion

Human body size has shown remarkable variation throughout our evolutionary history. Widespread global distribution has exposed human populations to a variety of environments that have been exploited for food resources in manifold ways. The elaboration of culture in the broad sense and cultures, as successful adaptive entities, has expanded both the environment and the selective pressures to which individuals have been exposed throughout their lives. Human physiological, anatomical, and developmental plasticity contribute substantially to variation in adult body size, that is, body sizes in which some population averages may show statures that are one-and-a -half and body weights that are twice that of other populations. These major differences in average individual sizes, however, have strong genetic components that are expressed through variable nutritional intakes and hormonal regulations, and are manifested by different growth rates and durations.

Darwinian selection has operated in favor of large body size to enhance fertility in women and provide for successful competition for mates in men. But large size in men also is desireable in the context of intra- and inter-population conflicts, which have been common throughout human history (132). Within technologically advanced societies large men and large women have higher fertility than smaller men and women, and taller men tend to be more successful economically and in leadership positions. On the other hand, in traditional or small-scale societies (that have characterized much of our evolutionary history), the energy cost of reproduction and producing large infants, children, and adults is often more than can be sustained by existing resources. In these cases, the selective pressures favoring large body size may be countered by selection for smaller body size and balances are achieved. With Late Paleolithic technology, a large human body size might be less favorable than a smaller body size when hunting for large mammals is conducted over long distances. And among the short-statured Ju/"hoansi, successful hunters tend to have higher fertility than their less-successful cohorts (133).

Life history theory is a productive way to integrate information about these opposing selective pressures to achieve optimal body sizes for individual populations (134). This idea to optimize the adaptive roles of population survival and population persistence through the major variables of individual maintenance, growth, reproduction, and longevity (survival to completed reproduction and beyond) is an appropriate way to explore these relationships.

## Author Contributions

The author confirms being the sole contributor of this work and has approved it for publication.

### Conflict of Interest

The author declares that the research was conducted in the absence of any commercial or financial relationships that could be construed as a potential conflict of interest.
